# Caffeine Modulates Vesicle Release and Recovery at Cerebellar Parallel Fibre Terminals, Independently of Calcium and Cyclic AMP Signalling

**DOI:** 10.1371/journal.pone.0125974

**Published:** 2015-05-01

**Authors:** Katharine L. Dobson, Claire Jackson, Saju Balakrishnan, Tomas C. Bellamy

**Affiliations:** 1 School of Life Sciences, University of Nottingham, Medical School, Queen's Medical Centre, Nottingham, United Kingdom; 2 Laboratory for Molecular Signalling, Babraham Institute, Babraham, Cambridge, United Kingdom; 3 Institute of Neuro & Sensory Physiology, Humboldtallee-23, Goettingen, Germany; University of Sydney, AUSTRALIA

## Abstract

**Background:**

Cerebellar parallel fibres release glutamate at both the synaptic active zone and at extrasynaptic sites—a process known as ectopic release. These sites exhibit different short-term and long-term plasticity, the basis of which is incompletely understood but depends on the efficiency of vesicle release and recycling. To investigate whether release of calcium from internal stores contributes to these differences in plasticity, we tested the effects of the ryanodine receptor agonist caffeine on both synaptic and ectopic transmission.

**Methods:**

Whole cell patch clamp recordings from Purkinje neurons and Bergmann glia were carried out in transverse cerebellar slices from juvenile (P16-20) Wistar rats.

**Key Results:**

Caffeine caused complex changes in transmission at both synaptic and ectopic sites. The amplitude of postsynaptic currents in Purkinje neurons and extrasynaptic currents in Bergmann glia were increased 2-fold and 4-fold respectively, but paired pulse ratio was substantially reduced, reversing the short-term facilitation observed under control conditions. Caffeine treatment also caused synaptic sites to depress during 1 Hz stimulation, consistent with inhibition of the usual mechanisms for replenishing vesicles at the active zone. Unexpectedly, pharmacological intervention at known targets for caffeine—intracellular calcium release, and cAMP signalling—had no impact on these effects.

**Conclusions:**

We conclude that caffeine increases release probability and inhibits vesicle recovery at parallel fibre synapses, independently of known pharmacological targets. This complex effect would lead to potentiation of transmission at fibres firing at low frequencies, but depression of transmission at high frequency connections.

## Introduction

Cerebellar parallel fibres form excitatory synapses with Purkinje neurons that exhibit facilitation during paired pulse stimulation. This phenomenon has been attributed to summation of calcium influx in the presynaptic terminals leading to an increase in release probability for the second pulse in the pair [1]. In addition to this form of short-term plasticity, release probability can also be increased by activation of presynaptic cAMP signalling pathways, resulting in PKA-dependent phosphorylation of several components of the presynaptic release machinery (principally, Rim1α and Rab3A), and PKA-independent activation of Epac, which collectively promote vesicle docking and priming [2–4]. These, and other, signalling pathways have been linked to presynaptic forms of long-term plasticity, most notably LTP during stimulation at 4–8 Hz [5–7].

In addition to release at the synaptic cleft, parallel fibre terminals also exhibit ectopic release—that is, fusion of vesicles outside of the active zone—releasing glutamate directly into the extracellular space [8,9]. This process mediates neuron-glial transmission, through the activation of Ca^2+^-permeable AMPA receptors on the Bergmann glia that enclose the synapses [10,11]. It has previously been shown that paired pulse facilitation of ectopic transmission is even more pronounced than synaptic transmission [12,13], but conversely, ectopic release also shows long-term depression at stimulation frequencies in the 0.1–1 Hz range, conditions under which synaptic transmission is potentiated [14]. The basis of this depression is the depletion of vesicles from ectopic sites [15], suggesting a deficit in the signalling processes linked to recycling of vesicles to docking sites [16,17].

We hypothesized that ectopic and synaptic sites may differ in their sensitivity to calcium release from internal stores, given that calcium has been implicated increasing vesicle recycling rate [18]. In investigating the effects of different calcium mobilizing agents, we discovered that the ryanodine receptor agonist, caffeine, has two striking effects on transmission at parallel fibre terminals. We show that, unexpectedly, these effects of caffeine do not depend on known pharmacological targets linked to calcium or cAMP signalling, and so conclude that a previously unrecognized pharmacological action of caffeine is exerted on presynaptic release at both synaptic and ectopic sites.

## Materials and Methods

### Animals

Rats (age 16–20 days) were humanely killed by cervical dislocation. All experiments were performed according to policies on the care and use of laboratory animals of British Home Office and European Community laws. The University of Nottingham Animal Welfare and Ethical Review Body approved the experiments. All efforts were made to minimize animal suffering and reduce the number of animals used.

### Cerebellar slice preparation

Transverse cerebellar slices (300 μm) were prepared from 16- to 20-day old Wistar rats of either sex, as previously described [19]. Briefly, rats were humanely killed by cervical dislocation, decapitated, and the cerebellum rapidly excised and sliced using a vibrating microtome (Leica VT1000S). For recording, slices were transferred to an immersion chamber and perfused with a solution containing (mM): NaCl (126), KCl (3), NaH_2_PO4 (1.2), NaHCO_3_ (25), glucose (15), MgSO_4_ (2), and CaCl_2_ (2) and continuously bubbled with carbogen (95% O_2_, 5% CO_2_). For Purkinje neuron experiments, the bath solution was supplemented with 20 μM picrotoxin to inhibit GABA_A_ receptors.

### Electrophysiology

Borosilicate recording electrodes were manufactured as previously described [19]. Internal solution consisted of (mM): K-gluconate (110), KCl (5), HEPES (50), EGTA (0.05), MgSO_4_ (4), ATP (4), GTP (0.2), phosphocreatine (9), and pH to 7.4 with 1 M KOH. Whole-cell voltage clamp recordings were made from Bergmann glia (holding potential -80 mV) and Purkinje neuron (holding potential -70 mV) somata in the Purkinje cell layer. Currents were low pass filtered at 4–5 kHz and sampled at 25 kHz, using Spike2 software (CED, Cambridge, UK). Series resistances ranged from 5 to 15 MΩ and were compensated by >85% in Purkinje neuron recordings, but uncompensated in glial recordings.

Parallel fibres were stimulated with a patch electrode (~1–2 MΩ) filled with bath solution and positioned in the molecular layer, connected to an isolated constant current stimulator (6.5–90 μA, 80 μs; Digitimer, Welwyn Garden City, UK). Stimulus was delivered as a pair of pulses with a 100 ms interval at a frequency of either 0.033 Hz or 1 Hz.

### Materials

Caffeine, dantrolene, and IBMX (3-isobutyl-1-methylxanthine) were purchased from Sigma-Aldrich (Gillingham, Dorset, UK). 2-APB (2-aminoethoxydiphenylborane), CGP 52432, DPCPX (8-cyclopentyl-1,3-dipropylxanthine), MPPG ((RS)-α-methyl-4-phosphonophenylglycine), cytochalasin D, staurosporine, ML-9 hydrochloride, picrotoxin, ryanodine, and thapsigargin were purchased from Tocris Bioscience (Bristol, UK). BAPTA (1,2-bis(2-aminophenoxy)ethane-N,N,N',N'-tetraacetic acid) and rimonabant hydrochloride were purchased from VWR International (Lutterworth, Leicestershire, UK). Forskolin was purchased from Fisher Scientific (Loughborough, Leicestershire, UK). Stock solutions of rimonabant, cytochalasin D, staurosporine, ML-9, ryanodine, and DPCPX were prepared in DMSO, with a final DMSO concentration of less than 0.1%. All other drugs were dissolved directly into the bath solution.

### Data analysis

Extrasynaptic current (ESC) and excitatory post-synaptic current (EPSC) traces shown are the average of five sequential recordings at the indicated frequency, unless otherwise indicated. Stimulus artefacts are truncated for clarity. Aggregate data are the mean ± s.e.m. of multiple cells as indicated in figure legends. Where stimulation frequency has been raised to 1 Hz, only every thirtieth stimulus is shown, to aid clarity. Decay time was measured as the time for an ESPC to decline from 90 to 10% of the peak current. Statistical significance of normalized data was tested for by single sample *t* test, except multiple comparisons, which were tested using one-way ANOVA followed by Dunnett's test. Differences were considered significant if P < 0.05.

To determine concentration dependence, data were fitted with a modified form of the Hill equation (where *R* = response amplitude, and *n*_*H*_ is the Hill coefficient):

$$y=\frac{(R_{\max}-R_{\min})x^{n_{H}}}{EC_{50}{}^{n_{H}}+x^{n_{H}}{}_{}}+R_{\min}$$

## Results

Synaptic and ectopic release sites exhibit differences in short-term plasticity with paired pulse stimulation, and in the ability to sustain transmission at baseline frequencies >0.1 Hz [13,14]. We therefore tested the effects of caffeine on paired pulse facilitation at 0.033 Hz and 1 Hz, to explore whether differential effects were observed at the two sites.

### Effects of caffeine on parallel fibre to Purkinje neuron transmission

**Fig 1:**
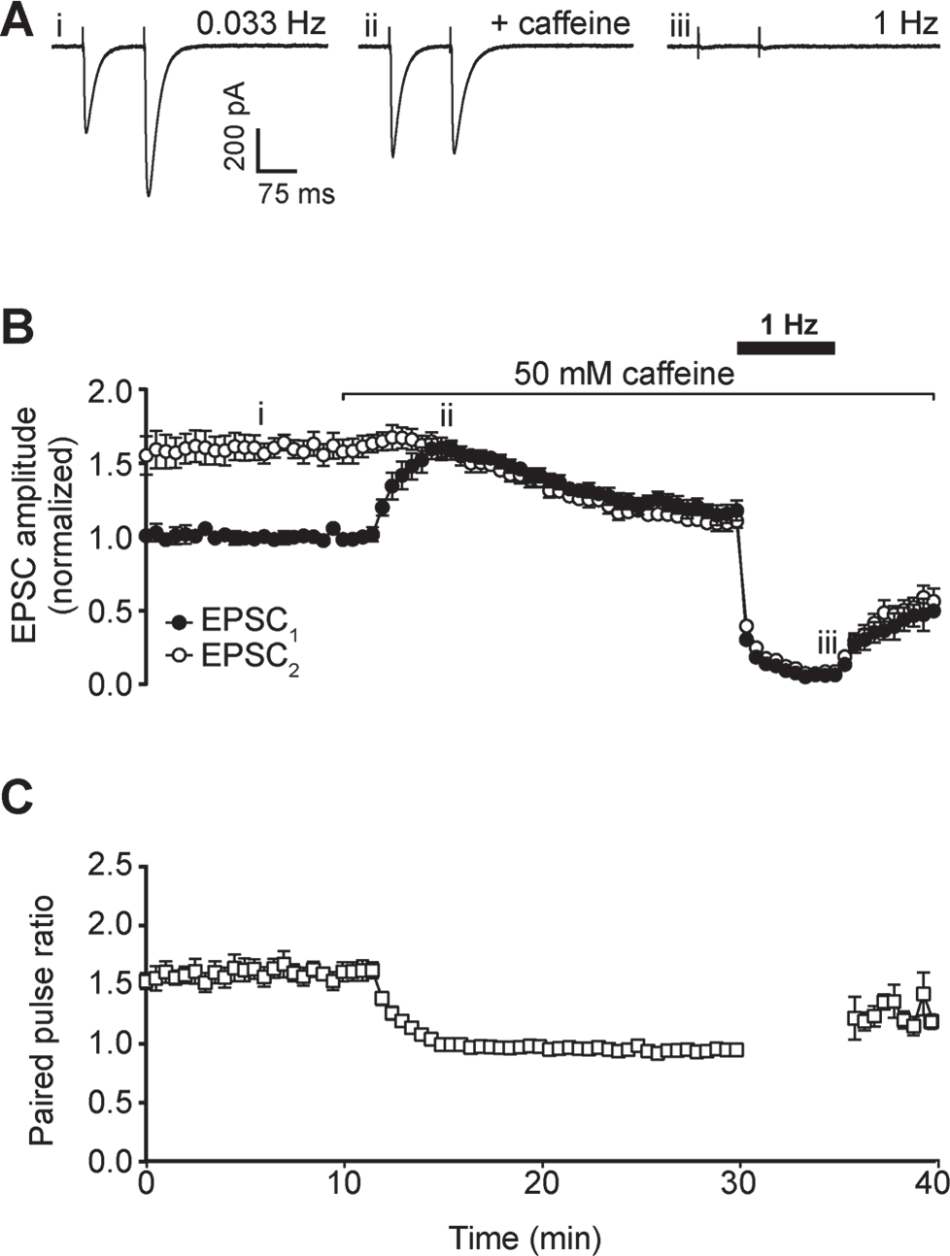
Effect of 50 mM caffeine on parallel fibre-Purkinje neuron signalling. A) Representative whole-cell recordings of neuronal excitatory post-synaptic currents (EPSC) generated by paired pulse stimulation (100 ms interval) of parallel fibres at 0.033 Hz before (first panel) and after (second panel) addition of 50 mM caffeine. Raising stimulation frequency to 1 Hz (third panel) for 5 min leads to depression of EPSC. Roman numerals refer to time points in 1B. B) Time course of caffeine effect on amplitude of the first (filled circles, EPSC_1_) and second (open circles, EPSC_2_) pulse in each pair after addition to bath. Note that during stimulation at 1 Hz mean values are only shown for every 30 s, to aid clarity. C) Time course of caffeine effect on mean paired pulse ratio. Values during 1 Hz stimulation are blanked due to negligible amplitude of both EPSCs. Data are mean ± s.e.m. from 6 cells (3 animals). Changes in mean amplitude of EPSC_1_ and paired pulse ratio at 15 min were statistically significant; P = 0.0003 and P <0.0001 respectively, single sample *t* test.

Parallel fibres were stimulated with a pair of pulses (100 ms interval) at a baseline frequency of 0.033 Hz. Under these control conditions, a pair of excitatory postsynaptic currents (EPSCs) are evoked in the Purkinje neuron which exhibit paired-pulse facilitation (Fig 1A and 1B). Perfusing the slice with 50 mM caffeine, a concentration that should maximally activate ryanodine receptors in the slice preparation (EC_50_ ~6 mM; [20]), increased the amplitude of the response to the first pulse (EPSC_1_) by 1.60 ± 0.07 fold. This increase peaked after 5 min of treatment, but thereafter EPSC_1_ amplitude declined progressively over the following 10–15 min.

In contrast to the increase in EPSC_1_, the amplitude of the response to the second pulse (EPSC_2_) did not change on addition of caffeine, so that the mean paired-pulse ratio fell to 0.99 ± 0.04 after 5 min of caffeine treatment (Fig 1C). Paired pulse ratio remained the same during the decline in EPSC_1_ amplitude after the peak, as both pulses decreased in parallel (Fig 1A and 1B).

In addition to the changes in EPSC amplitude, 50 mM caffeine also caused a small but statistically significant increase in decay time for both responses in the pair (EPSC_1_ control decay = 34.30 ± 1.37 ms, EPSC_1_ caffeine decay = 46.29 ± 8.04 ms; EPSC_2_ control decay = 38.99 ± 1.32 ms, EPSC_2_ caffeine decay = 53.82 ± 6.81 ms, means ± s.e.m; n = 6; P = 0.0434 and P = 0.0047 respectively, single sample *t* test).

To test the effect of caffeine on transmission strength at higher stimulation frequencies, baseline frequency was raised to 1 Hz. This increase led to the near complete loss of the EPSC for both pulses, within 5 min (Fig 1A and 1B). After the stimulation frequency was returned to a baseline of 0.033 Hz, both EPSC amplitudes demonstrated a slow recovery over 5 min.

### Effects of caffeine on parallel fibre to Bergmann glial transmission

**Fig 2:**
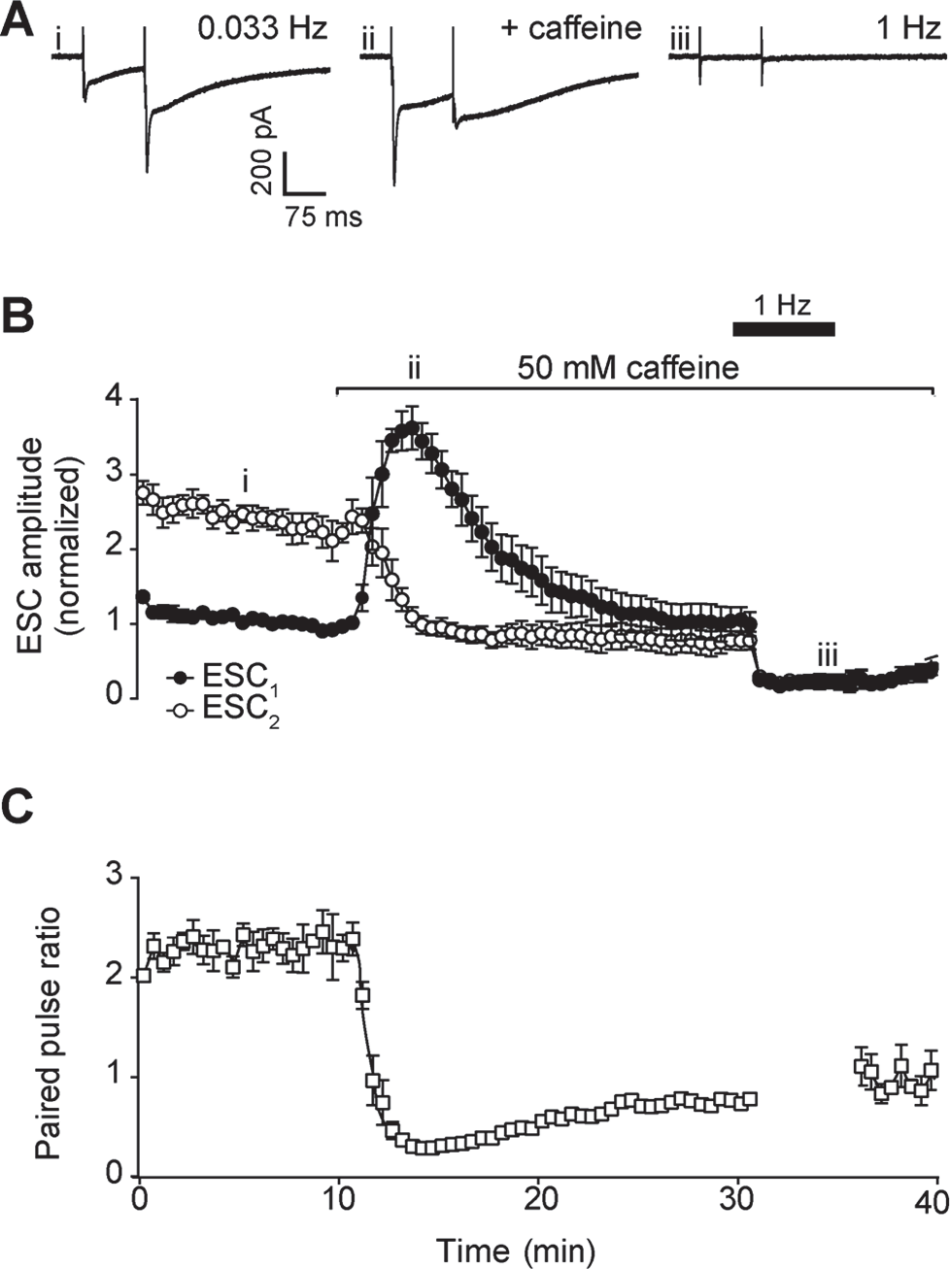
Effect of 50 mM caffeine on parallel fibre-Bergmann glia signalling. A) Representative whole-cell recordings of Bergmann glial extrasynaptic currents (ESC) generated by paired pulse stimulation (100 ms interval) of parallel fibres at 0.033 Hz before (first panel) and after (second panel) addition of 50 mM caffeine. Raising stimulation frequency to 1 Hz (third panel) for 5 min leads to depression of ESC. Roman numerals refer to time points in 2B. B) Time course of caffeine effect on amplitude of the first (filled circles, ESC_1_) and second (open circles, ESC_2_) pulse in each pair after addition to bath. C) Time course of caffeine effect on mean paired pulse ratio. Data are mean ± s.e.m. from 6 cells (4 animals). Changes in mean amplitude of ESC_1_ and paired pulse ratio at 15 min were statistically significant; P = 0.0036 and P = 0.0002 respectively, single sample *t* test.

Whole cell recording of extrasynaptic currents (ESCs) in Bergmann glial cells allows the measurement of ectopic release from parallel fibre terminals, due mainly to activation of glial AMPA receptors and glutamate transporters [11,13]. Under the same stimulation conditions as Purkinje neuron recordings, the amplitude of ESC_1_ was greatly enhanced by application of 50 mM caffeine, with mean fold change of 3.06 ± 0.25 after 5 min (Fig 2A and 2B). As with Purkinje neuron responses, this enhancement was not sustained, and amplitude declined progressively over 10–15 min to return to pre-treatment levels.

In contrast to Purkinje neurons, the amplitude of ESC_2_ in Bergmann glia was rapidly reduced within 5 min of caffeine addition, from 2.11 ± 0.27 to 0.96 ± 0.12 of control ESC_1_ amplitude, and remained stable for a further 15 min (Fig 2A and 2B). Accordingly, mean paired-pulse ratio fell dramatically to 0.31 ± 0.03 after 5 min of caffeine treatment (Fig 2C).

The decay time of Bergmann glial ESCs was not significantly altered by caffeine treatment when stimulating at 0.033 Hz (ESC_1_ control decay = 78.73 ± 2.18 ms, ESC_1_ caffeine decay = 93.32 ± 0.28 ms, ESC_2_ control decay = 99.05 ± 5.07 ms, ESC_2_ caffeine decay = 127.17 ± 15.21 ms; means ± s.e.m.; n = 6; P = 0.1255 and P = 0.3606 respectively, single sample *t* test).

Raising stimulation frequency to 1 Hz led to the complete loss of ESCs (Fig 2A and 2B), but this is in keeping with the response of ectopic transmission under normal conditions [19].

### Concentration-dependence of caffeine effects

Caffeine appeared to be having at least two distinct effects on transmission at the parallel fibre- Purkinje neuron synapse: an enhancement of EPSC amplitude for the first pulse in the pair, and a decrease in the ability of the terminal to sustain transmission at 1 Hz. We next investigated the concentration-dependence of these two effects.

**Fig 3:**
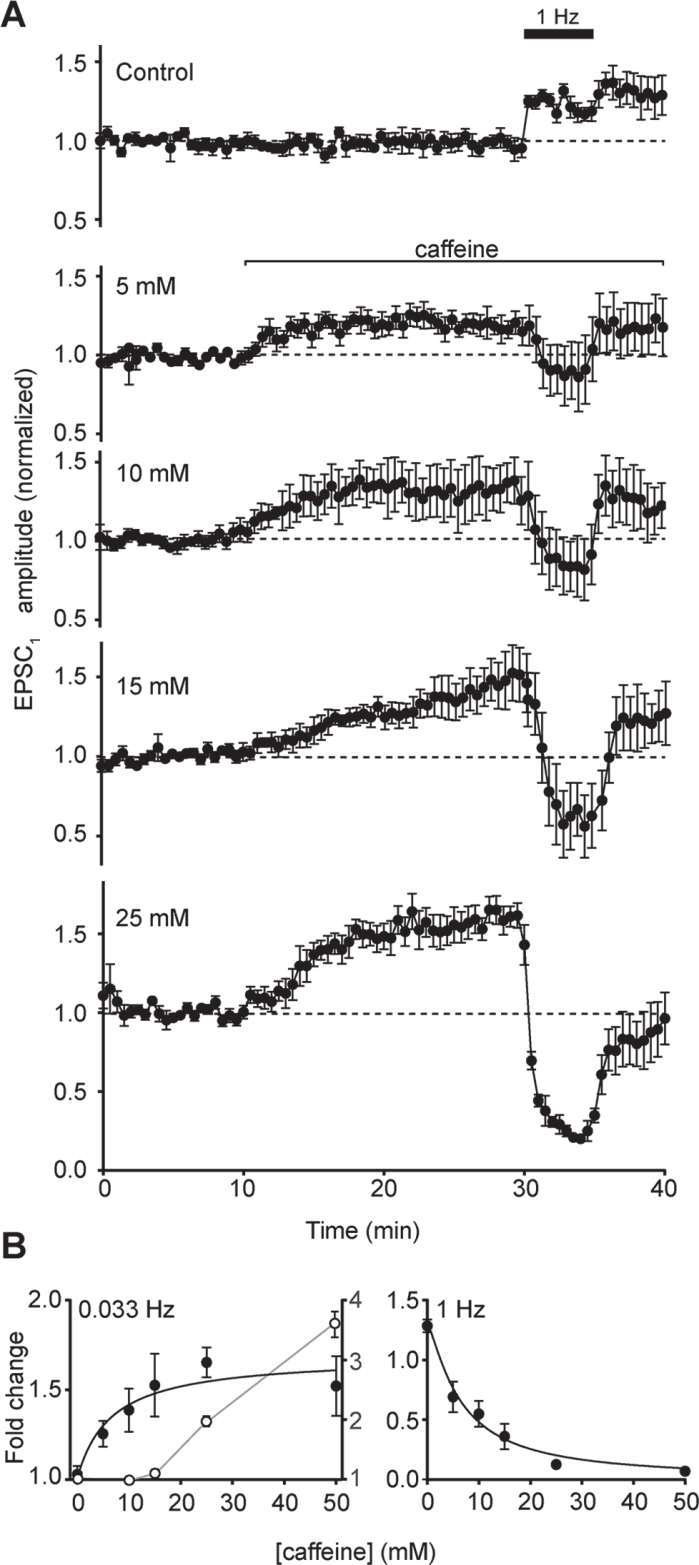
Concentration-dependent effects of caffeine on parallel fibre-Purkinje neuron signalling. A) Mean amplitude of Purkinje neuron EPSC_1_ for control cells, and a range of caffeine concentrations, during stimulation at 0.033 Hz and 1 Hz (as indicated). Data are mean ± s.e.m. from 5–6 cells (2–3 animals). B) Concentration response curves for maximal response to caffeine during 0.033 Hz stimulation (left panel), and maximal inhibition during 1 Hz stimulation (right panel). Data are mean ± s.e.m. Filled circles show Purkinje neuron EPSC (cells = 5–6, animals = 2–3), and open circles show Bergmann glial ESC (cells = 3–5, animals = 1–4). Purkinje neuron data were well fitted by the Hill equation (black lines), with EC_50_ of 6.50 mM at 0.033 Hz (n_H_ = 1) and IC_50_ of 6.55 mM at 1 Hz (n_H_ = -1.26). Hill equation could not be adequately fitted to glial ESC data.

Caffeine increased the amplitude of EPSC_1_ at 0.033 Hz, and decreased the amplitude at 1 Hz, in a concentration-dependent manner (Fig 3A and 3B). At the highest concentration tested (50 mM) the increase in EPSC_1_ amplitude reached a peak before declining (Fig 1B), but at lower concentrations, the increase was sustained over 10 min (Fig 3A). In contrast to the effects on amplitude, no significant changes in decay time were detected for either EPSC_1_ or EPSC_2_ at any caffeine concentration <50 mM (P > 0.05 at all concentrations by single sample *t* test).

Plotting the maximum and minimum amplitudes attained at the different baseline frequencies allows potency to be determined (Fig 3B). For potentiation at 0.033 Hz, EC_50_ = 6.55 mM. For depression at 1 Hz, IC_50_ = 6.50 mM. These values are closely similar to the potency of caffeine as a ryanodine receptor agonist [20].

The concentration dependence of the enhancement of Bergmann glial ESC_1_ was also measured (Fig 3B). A concentration-dependent increase in ESC amplitude was observed, but the data were poorly fitted by the Hill equation, and no maximum response could be determined (Fig 3B). Estimating the EC_50_ for the enhancement of glial ESC_1_ based on the response at 50 mM gave a value of >28 mM (Fig 3B), indicating a lower potency for caffeine at ectopic than synaptic sites.

### Effect of inter-stimulus interval on paired pulse ratio in the presence of caffeine

To characterize the effect of caffeine on facilitation more fully, we explored how inter-stimulus interval affected paired pulse ratio before and after treatment.

**Fig 4:**
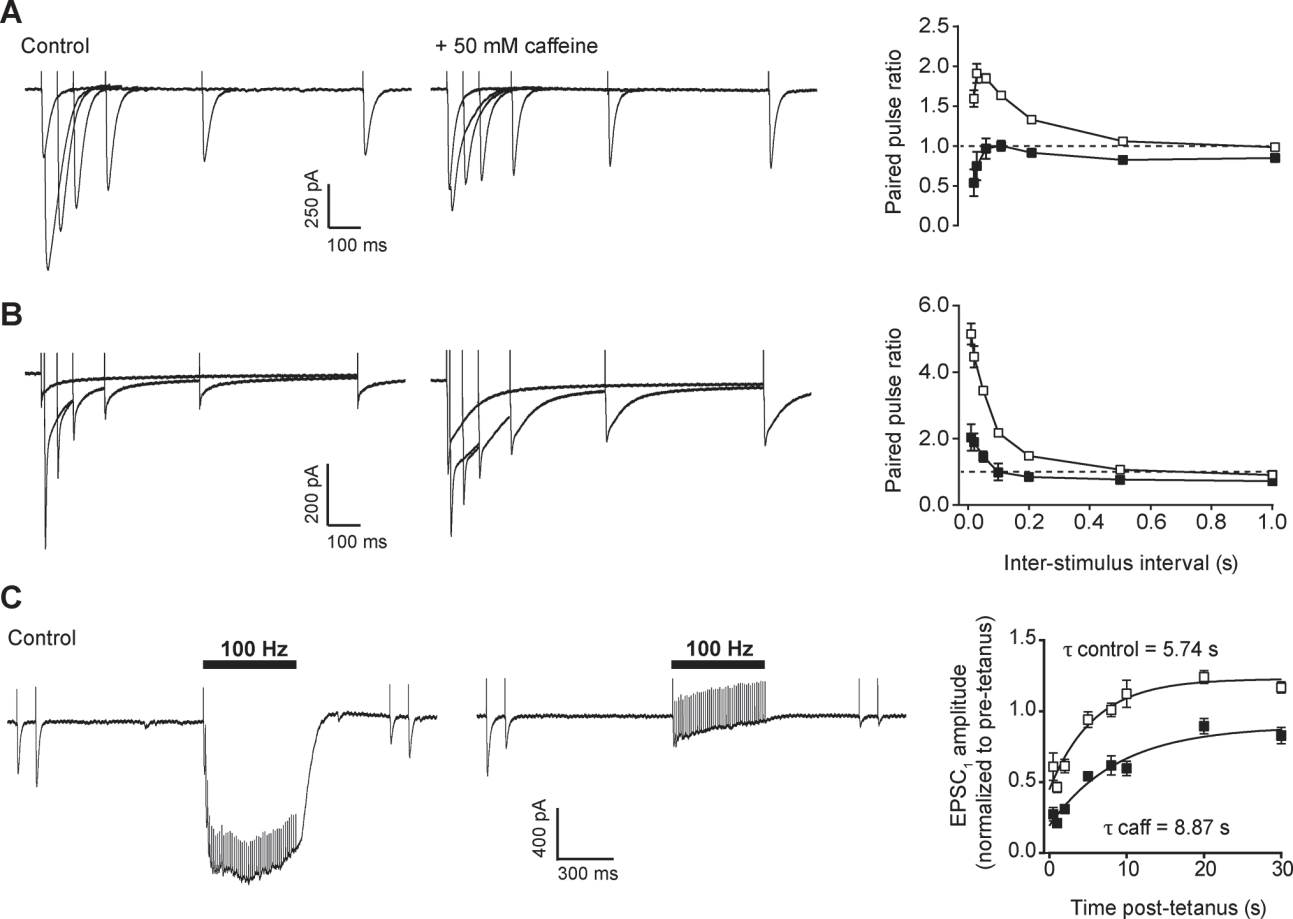
Effect of inter-stimulus interval on paired-pulse facilitation and post-tetanic recovery in the presence and absence of 50 mM caffeine. A) Overlaid representative whole-cell recordings of neuronal excitatory post-synaptic currents (EPSC) generated by paired pulse stimulation with inter-stimulus intervals of 0.01, 0.05, 0.1, 0.2, 0.5, and 1 second, before (left hand panel) and after (centre panel) addition of 50 mM caffeine. Right hand panel shows the relationship between inter-stimulus interval and paired pulse ratio for control (open squares) and caffeine treated (filled squares) neurons. Data are mean ± s.e.m. for 7 cells (2 animals), with EPSC amplitude being defined as the difference between peak inward current and current immediately before stimulus (note that this may underestimate EPSC_2_ amplitude for very short intervals). B) Bergmann glial cell extrasynaptic currents under the same conditions as panel A. Data are mean ± s.e.m. for 4 cells (2 animals). C) Representative whole-cell recording of neuronal EPSCs before, during and after tetanic stimulation. First stimulus pair was delivered 1 s prior to a tetanus (100 Hz, 50 pulses) with a further stimulus pair delivered 0.5 s after the tetanus. Recordings are shown from the same cell before (left hand panel) and after addition of 50 mM caffeine (centre panel) to bath solution. Right hand panel shows mean EPSC_1_ amplitude against post-tetanic recovery time in control conditions (open squares) and in the presence of 50 mM caffeine (filled squares). Data are mean ± s.e.m. for 9 cells (4 animals). Solid lines are exponential fits. Slices were incubated with 2 μM DPCPX, 10 μM CGP52432, 100 μM MPPG, and 5 μM rimonabant to inhibit presynaptic inhibitory receptors.

For untreated Purkinje neurons, short intervals (10–20 ms) exhibited maximal paired pulse facilitation of EPSCs, which declined in magnitude with increasing pulse interval, until no facilitation was evident at 1 s (Fig 4A). This is consistent with other reports [12,21,22]. In contrast, after incubation with 50 mM caffeine, paired pulse facilitation was effectively abolished. Mean paired pulse ratio was less than or equal to 1 at all intervals tested (Fig 4A), reflecting the substantial increase in amplitude of EPSC_1_ with no corresponding increase in amplitude of EPSC_2_. This reversal of facilitation to depression was most evident at short intervals.

For parallel fibre to Bergmann glial transmission, paired pulse facilitation is more pronounced than parallel fibre to Purkinje neuron transmission, and facilitation decreases with increasing pulse interval (Fig 4B). After treatment with caffeine, paired pulse ratio is reduced at all intervals, although in contrast to Purkinje neuron EPSCs, facilitation is still observed at very short intervals (Fig 4B).

### Tetanic stimulation

To investigate the effects of caffeine on short-term plasticity in more detail, we stimulated parallel fibres at 100 Hz for 50 pulses.

Bergmann glial ESCs depress during tetanic stimulation, and recovery post-tetanus is impaired [15]. Consequently, we focussed on the effects of caffeine on synaptic transmission. To limit activation of presynaptic neuromodulatory receptors (A1R, GABA_B_R, mGluR4, CB1R), we pre-incubated the slice with a cocktail of antagonists (see Fig 4 legend), to block G_i/o_ inhibitory pathways that can reduce release probability [23–27].

Consistent with our previous study [15] a tetanus of 50 pulses at 100 Hz led to failure of transmission, presumably due to exhaustion of the readily releasable pool, as the majority of this current is attributable to postsynaptic AMPA receptor activation (Fig 4C). After the tetanus, the recovery of EPSC amplitude has a biphasic time course, with a rapid recovery of 61% ± 10% of amplitude within 0.5 s, followed by a slower, exponential recovery of the remaining EPSC amplitude (τ = 5.74 s) with evidence of an overshoot above pre-tetanus amplitude (Fig 4C).

After treatment with caffeine, three changes in transmission are evident. First, there is a dramatic decrease in the current generated during the tetanus, with an apparent failure of transmission within 3–5 pulses (Fig 4C). Second, the fast recovery phase post-tetanus is reduced relative to control conditions (28% ± 5% of pre-tetanus amplitude). Third, the exponential recovery phase is slowed (τ = 8.87 s) and does not reach pre-tetanus levels within the 30 s investigated (Fig 4C). This pattern of synaptic transmission during caffeine treatment is closely similar to neuron-glial transmission at ectopic sites [15], suggesting that caffeine has compromised vesicle recovery mechanisms in the active zone, mimicking behaviour normally observed at ectopic release sites.

### Inhibiting calcium release from stores does not alter caffeine effects

Having characterized the consequences of caffeine treatment for synaptic and ectopic transmission, we next sought to identify the targets of caffeine that were responsible for the observed effects.

Caffeine is known to affect calcium release from internal stores through two mechanisms: agonism of ryanodine receptors (EC_50_ ~ 6 mM; [20]) and antagonism of InsP_3_ receptors (IC_50_ ~ 1.64 mM; [28] although see also [29]). Given that calcium signalling is required for both vesicle release and recycling [18], it is plausible that modification of cytosolic calcium levels by disruption of release from stores could account for the observed effects of caffeine. Contrary to this hypothesis, existing evidence suggests that store release does not have a significant effect on EPSC amplitude or paired pulse facilitation at parallel fibre synapses [30,31]. Nevertheless, the effect of caffeine on transmission at 1 Hz may reflect disruption of intra-terminal calcium signalling.

**Fig 5:**
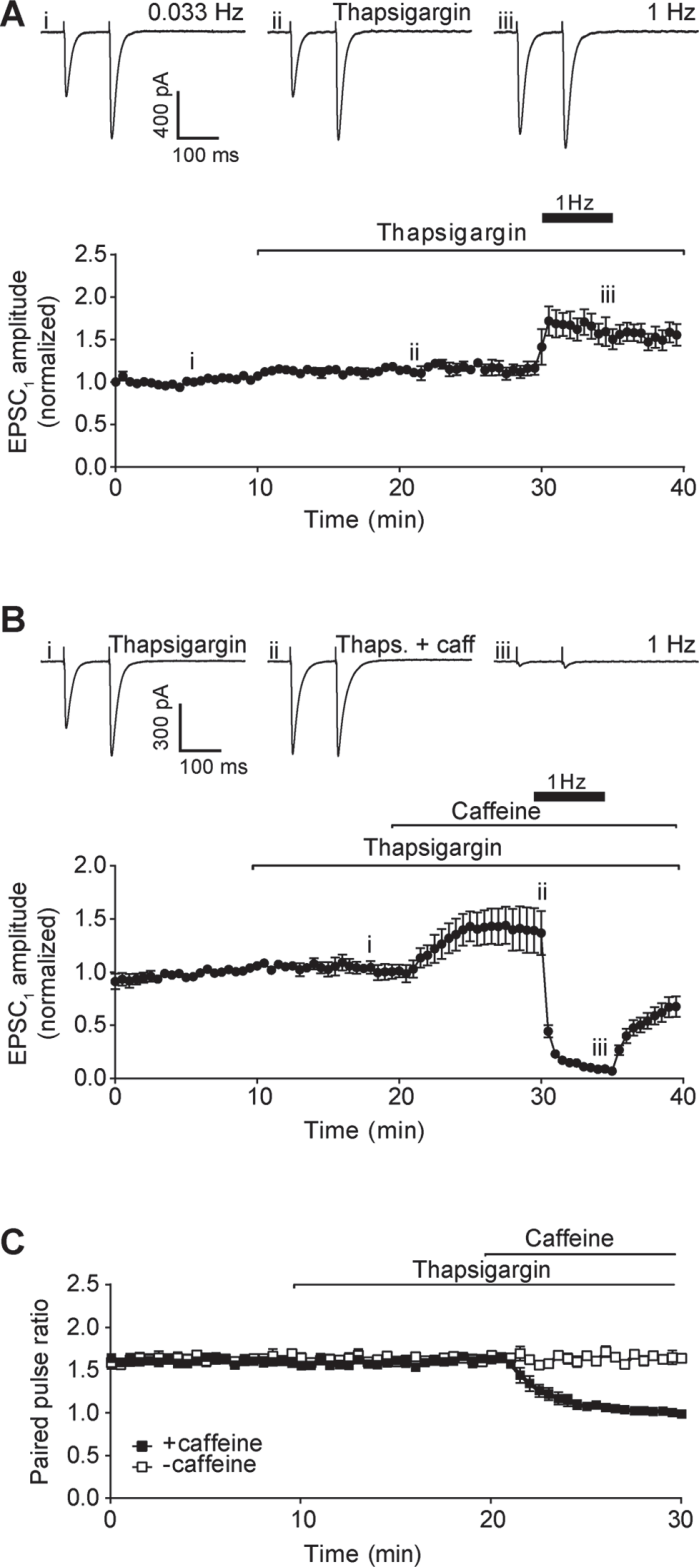
Inhibition of SERCA pump. A) Representative recordings of Purkinje neuron EPSC under control conditions (upper left panel), after addition of 2 μM thapsigargin (upper centre panel), and after raising stimulation frequency to 1 Hz for 5 min (upper right panel). Roman numerals refer to time points in 5A (lower panel). Lower panel shows time course of thapsigargin effect on Purkinje neuron EPSC_1_ amplitude. Data are mean ± s.e.m. from 6 cells (3 animals). B) Representative recordings of Purkinje neuron EPSC after addition of 2 μM thapsigargin (upper left panel), after addition of 50 mM caffeine in the presence of thapsigargin (upper centre panel), and following 5 min of 1 Hz stimulation (upper right panel). Roman numerals refer to time points in 5B (lower panel). Lower panel shows time course of thapsigargin and caffeine treatment on Purkinje neuron EPSC_1_ amplitude. Data are mean ± s.e.m. from 7 cells (4 animals). C) Time course of paired pulse ratio at 0.033 Hz during thapsigargin treatment in the absence (open squares) and presence of caffeine (filled squares).

To investigate this hypothesis, we first depleted store calcium levels with the sarco/endoplasmic reticulum Ca^2+^-ATPase (SERCA) inhibitor, thapsigargin (2 μM). Bath application of 2 μM thapsigargin alone had no effect on EPSC amplitude, paired pulse ratio, or potentiation at 1 Hz (Fig 5A), consistent with previous results [31]. Co-application of thapsigargin and caffeine (50 mM) led to a similar result to caffeine alone: an increase in EPSC_1_ amplitude and concomitant decrease in paired pulse ratio, and a loss of transmission at 1 Hz (Fig 5B and 5C).

Depletion of stores may have an unpredictable effect on cytoplasmic calcium levels due to the activation of store-operated calcium entry (SOCE) in the presence of the extracellular calcium concentration necessary for synaptic transmission. We therefore tested the hypothesis further with the use of a ryanodine receptor antagonist (dantrolene, 20 μM; [32]), and an inhibitor of InsP_3_ receptors and SOCE channels (2-APB, 10 μM; [33]) We tested all three inhibitors of store-dependent calcium signalling at submaximal (10 mM) and maximal (50 mM) concentrations of caffeine.

**Fig 6:**
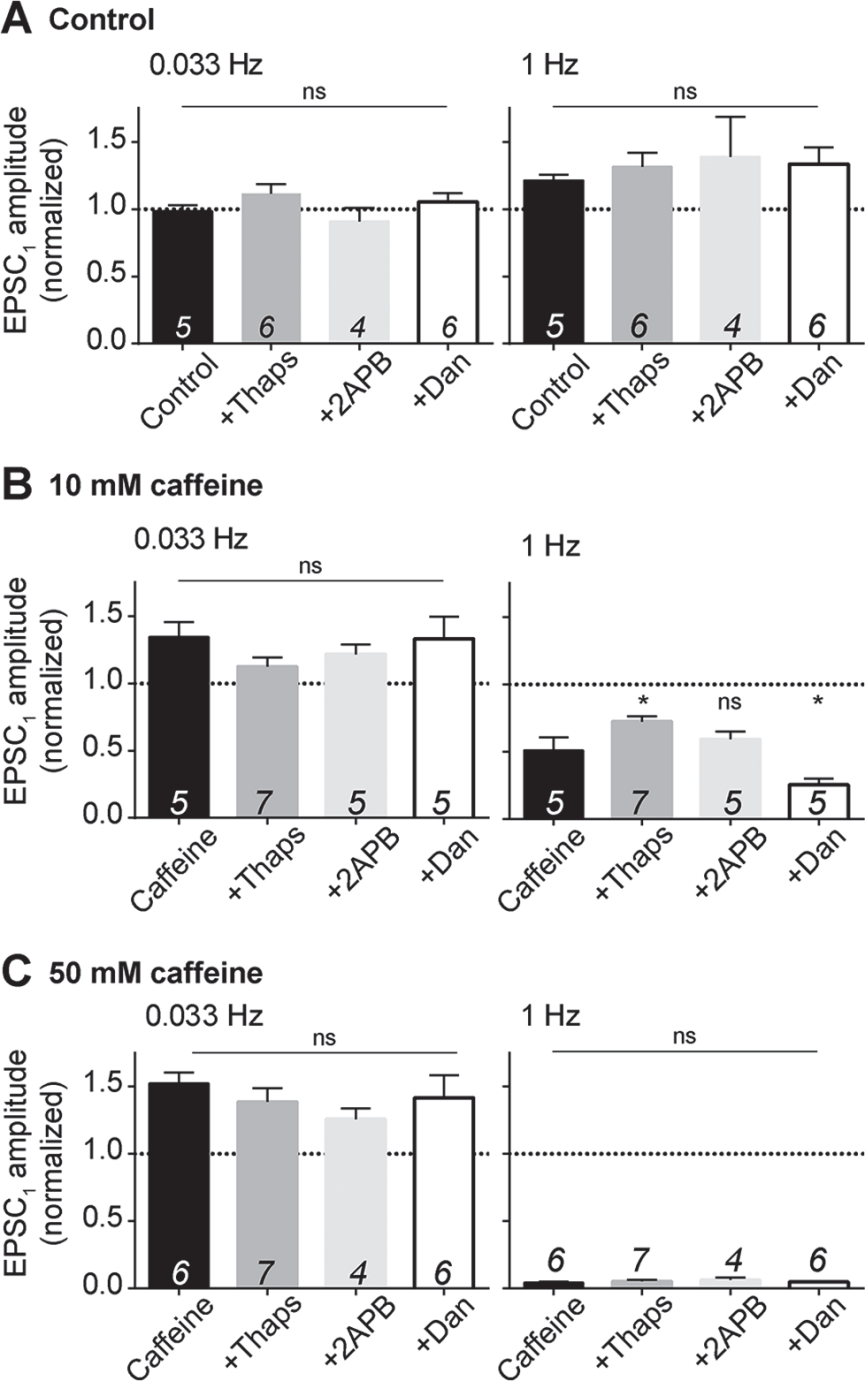
Modulation of calcium release from internal stores. A) Mean EPSC_1_ amplitude following incubation in the absence (control) and presence of the stated inhibitors (Thaps: thapsigargin, 2 μM; 2-APB, 10 μM; Dan: dantrolene, 20 μM) for 20 minutes during 0.033 Hz stimulation (left panel) and a subsequent 5 minutes with 1 Hz stimulation (right panel). See Fig 5A for representative full time course. B) Mean EPSC_1_ amplitude in control cells, or cells pre-incubated for 10 minutes with the stated inhibitors, following addition of 10 mM caffeine for a further 10 min. Mean values are for EPSC_1_ amplitude after 10 min stimulation in the presence of caffeine at 0.033 Hz (left panel) and a further 5 min stimulation at 1 Hz (right panel). C) As B, for cells exposed to 50 mM caffeine. Numbers superimposed over each bar on the chart are number of cells per group. All data sets are the result of experiments using slices harvested from a minimum of 2 animals, with the exception of 10 mM caffeine plus dantrolene, which is from 1 animal. Statistical significance of differences in mean between control and inhibitors, or caffeine in the presence and absence of inhibitors, was tested for using one-way ANOVA followed by Dunnett's multiple comparisons test. *P <0.05; ns, P >0.05. Single sample *t* tests were used to compare caffeine data to their respective controls: control vs 50 mM at 0.033 Hz—P = 0.0003; control vs 50 mM at 1 Hz—P < 0.0001; control vs 10 mM at 0.033 Hz—P = 0.0158; control vs 10 mM at 1 Hz—P = 0.0001.

None of the inhibitors had any statistically-significant effect of transmission when applied to the bath alone, at either 0.033 Hz or 1 Hz stimulation frequencies (Fig 6A). When co-applied with 10 mM caffeine, none of the inhibitors had a statistically significant effect on mean EPSC_1_ amplitude at 0.033 Hz, compared to caffeine alone (Fig 6B). Thapsigargin reduced the depression of transmission at 1 Hz relative to 10 mM caffeine alone (Fig 6B), though the mean amplitude was still significantly less than the same thapsigargin treatment in the absence of caffeine (P <0.003, unpaired *t* test; cf. Fig 6A). In contrast, dantrolene enhanced the depression at 1 Hz (Fig 6B), possibly implying an additive effect of these two compounds. At the higher caffeine concentration of 50 mM, where the effect size was greater, none of the inhibitors had any statistically significant effect on EPSC amplitude at 0.033 Hz or 1 Hz (Fig 6C).

Collectively, these results suggest that release of calcium from internal stores is unlikely to account for the effects of caffeine on synaptic transmission, with the inhibitors showing only minor and inconsistent modulation of effect size at 10 mM caffeine.

### Modulation of cAMP signalling does not alter caffeine effects

Caffeine inhibits multiple isoforms of phosphodiesterase (PDE), with the effect of increasing cAMP (and cGMP) levels [34]. Elevation of cAMP can increase release probability, through increased PKA-dependent phosphorylation of several accessory proteins in the presynaptic SNARE complex, and could therefore account for the observed effect of caffeine on EPSC amplitude. To test this hypothesis, we applied an alternative PDE inhibitor, isobutylmethylxanthine (IBMX, 1 mM), to determine whether it could reproduce and/or occlude the effects of caffeine.

**Fig 7:**
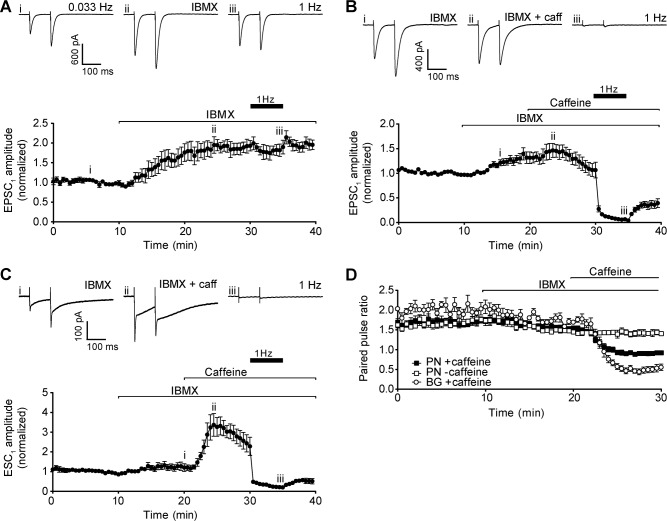
Inhibition of phosphodiesterase activity. A) Representative recordings of Purkinje neuron EPSC under control conditions (upper left panel), after addition of 1 mM IBMX (upper centre panel), and after raising stimulation frequency to 1 Hz for 5 min (upper right panel). Roman numerals refer to time points in 7A (lower panel). Lower panel shows time course of IBMX effect on Purkinje neuron EPSC_1_ amplitude. Data are mean ± s.e.m. from 5 cells (2 animals). B) Representative recordings of Purkinje neuron EPSC after addition of 1 mM IBMX (upper left panel), after addition of 50 mM caffeine in the presence of IBMX (upper centre panel), and following 5 min of 1 Hz stimulation (upper right panel). Roman numerals refer to time points in 7B (lower panel). Lower panel shows time course of IBMX and caffeine treatment on Purkinje neuron EPSC_1_ amplitude. Data are mean ± s.e.m. from 9 cells (4 animals). C) Representative recordings of Bergmann glial ESC after addition of 1 mM IBMX (upper left panel), after addition of 50 mM caffeine in the presence of IBMX (upper centre panel), and following 5 min of 1 Hz stimulation (upper right panel). Roman numerals refer to time points in 7C (lower panel). Lower panel shows time course of IBMX and caffeine treatment on glial ESC_1_ amplitude. Data are mean ± s.e.m. from 5 cells (2 animals). D) Time course of paired pulse ratio of synaptic transmission at 0.033 Hz during IBMX treatment in the absence (open squares) and presence of caffeine (filled squares), and ectopic transmission in the presence of caffeine (open circles).

Application of IBMX alone resulted in an increase in amplitude of EPSC_1_ at 0.033 Hz paired stimulation (Fig 7A). However, in contrast to caffeine, the amplitude of EPSC_2_ was also increased by IBMX, meaning that paired pulse ratio was decreased to a smaller extent than with caffeine, and facilitation was maintained (Fig 7D). Raising stimulation frequency to 1 Hz had no effect on either EPSC amplitude (Fig 7A), again distinguishing the effects of PDE inhibition from that of caffeine.

Addition of 50 mM caffeine to slices pre-treated with IBMX resulted in a small additional increase in mean EPSC_1_ amplitude, which was followed by a decline consistent with that observed with caffeine alone (Fig 7B). Concurrently, caffeine also caused a decrease in EPSC_2_ amplitude, and therefore caused the typical decrease in paired pulse ratio and loss of facilitation (Fig 7D).

The effects of PDE inhibition on the initial increase in EPSC_1_ amplitude evoked by caffeine are equivocal (Fig 7B), possibly because the magnitude in mean increase for EPSC_1_ is similar for IBMX and caffeine, and that the biphasic effect of caffeine on EPSC_1_ amplitude makes interpretation challenging. We therefore investigated the effect of IBMX on ectopic transmission, where the amplitude of ESC_1_ enhancement by caffeine is much more pronounced (Fig 2A and 2B).

Addition of 1 mM IBMX to the bath enhanced glial ESC_1_ to a similar extent as neuronal EPSC_1_, and as with synaptic transmission, paired pulse ratio was reduced but still showed facilitation (Fig 7C and 7D). Co-application of caffeine after pre-treatment with IBMX led to a dramatic further increase in mean ESC_1_ amplitude and reversal of paired pulse facilitation, on a similar scale to caffeine alone (Fig 2B), suggesting that IBMX and caffeine are working through different mechanisms to potentiate ESC_1_ amplitude.

**Fig 8:**
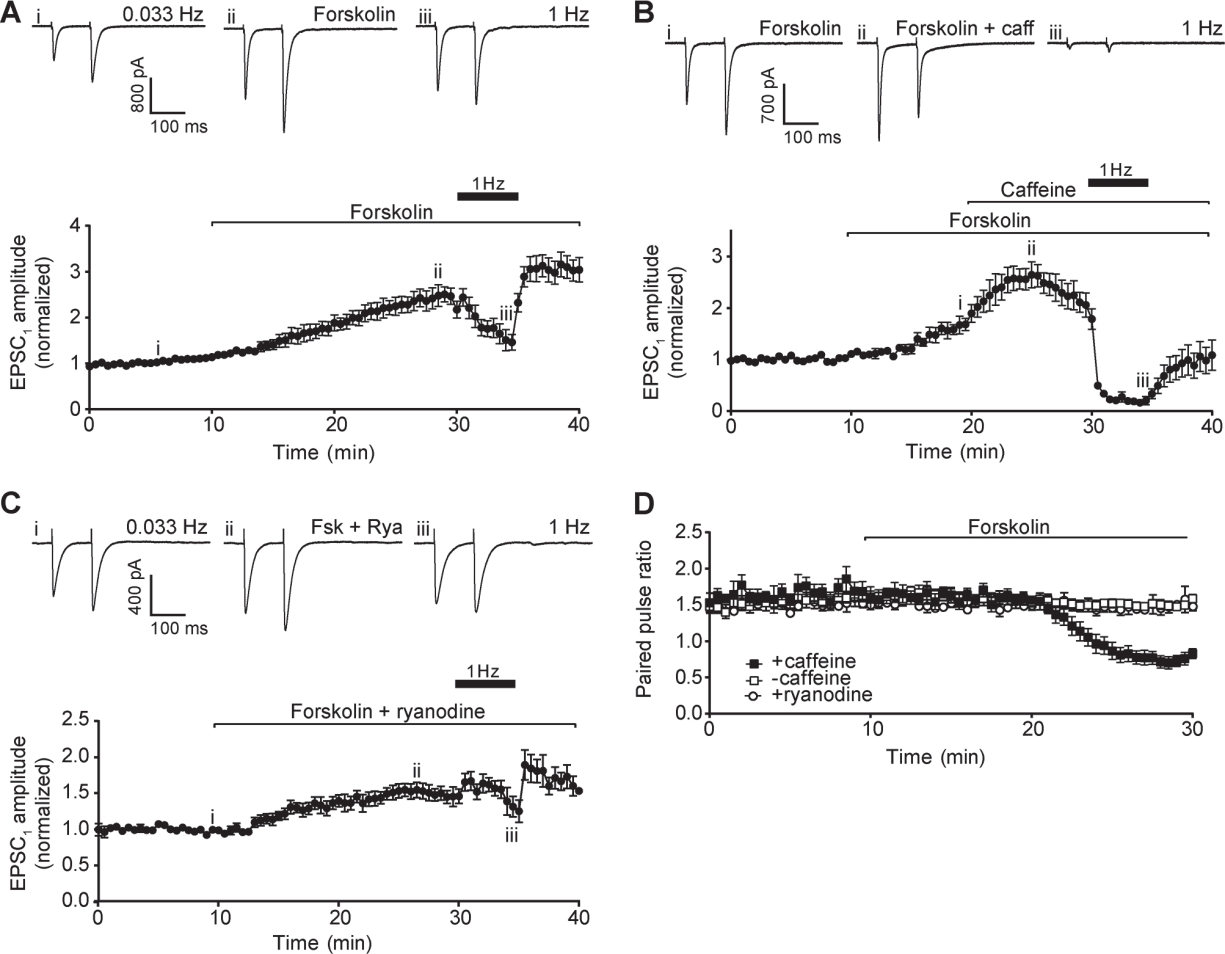
Activation of adenylyl cyclase. A) Representative recordings of Purkinje neuron EPSC under control conditions (upper left panel), after addition of 10 μM forskolin (upper centre panel), and after raising stimulation frequency to 1 Hz for 5 min (upper right panel). Roman numerals refer to time points in 8A (lower panel). Lower panel shows time course of forskolin effect on Purkinje neuron EPSC_1_ amplitude. Data are mean ± s.e.m. from 5 cells (3 animals). B) Representative recordings of Purkinje neuron EPSC after addition of 10 μM forskolin (upper left panel), after addition of 50 mM caffeine in the presence of forskolin (upper centre panel), and following 5 min of 1 Hz stimulation (upper right panel). Roman numerals refer to time points in 8B (lower panel). Lower panel shows time course of forskolin and caffeine treatment on Purkinje neuron EPSC_1_ amplitude. Data are mean ± s.e.m. from 5 cells (3 animals). C) Representative recordings of Purkinje neuron EPSC under control conditions (upper left panel), after addition of 500 nM ryanodine in the presence of forskolin (upper centre panel), and following 5 min of 1 Hz stimulation (upper right panel). Roman numerals refer to time points in 8C (lower panel). Lower panel shows time course of forskolin and ryanodine treatment on Purkinje neuron EPSC_1_ amplitude. Data are mean ± s.e.m. from 5 cells (2 animals). D) Time course of paired pulse ratio at 0.033 Hz during forskolin treatment in the absence (open squares) and presence of caffeine (filled squares), and in the presence of ryanodine (open circles).

To further test the potential role of cAMP signalling in the observed phenomena, we applied an activator of adenylyl cyclase to raise cAMP levels through a PDE-independent route. Forskolin (10 μM) was applied alone and with 50 mM caffeine. As with IBMX, forskolin increased Purkinje neuron EPSC_1_ and EPSC_2_ amplitudes at 0.033 Hz stimulation, with only a small decrease in paired pulse ratio (Fig 8A and 8D). Raising stimulation frequency to 1 Hz caused a decrease in mean EPSC_1_ amplitude, but to a lesser extent that that observed with caffeine (Fig 8A and 8D). Finally, forskolin did not block the effects of caffeine on paired pulse ratio nor the marked depression during 1 Hz stimulation (Fig 8B and 8D).

Collectively, these results show that enhancing release probability through cAMP dependent mechanisms cannot account for the effects of caffeine on paired pulse ratio, or depression during 1 Hz stimulation.

Finally, it is possible that the caffeine effect depends on modulation of both calcium and cAMP signalling pathways simultaneously. We therefore applied ryanodine at a concentration that is known to act as an agonist at the ryanodine receptor (500 nM; [35]) concurrently with 10 μM forskolin (Fig 8C and 8D). This combination gave a similar result to forskolin alone, although the addition of ryanodine reduced the magnitude of potentiation, and the extent of depression at 1 Hz (Fig 8C).

### Caffeine response does not require calcium release in the Purkinje neuron

Bath application of caffeine will act on all cell types and compartments, so we next sought to determine whether caffeine was acting within the Purkinje neuron, rather than the presynaptic terminals. Caffeine was added to the internal pipette solution at 15 mM—a concentration sufficient to provoke substantial changes in paired pulse ratio and depression at 1 Hz when applied to the bath (Fig 3A and 3B).

**Fig 9:**
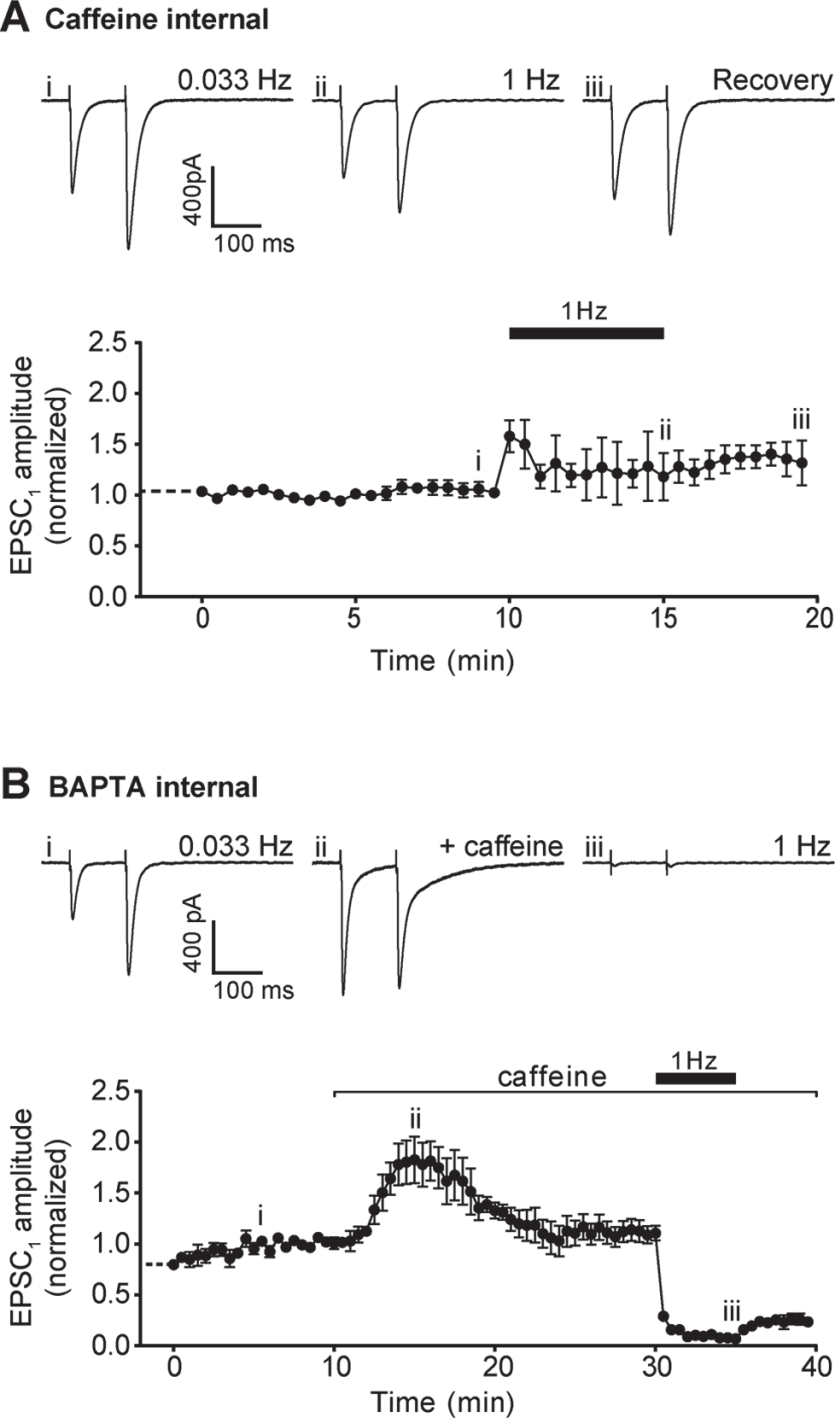
Caffeine response following modification of internal buffer solution. A) Representative recordings of Purkinje neuron EPSC with 15 mM caffeine in the internal pipette solution after 10 min of paired pulse stimulation at 0.033 Hz (upper left panel), after a further 5 min stimulation at 1 Hz (upper centre panel), and following a 5 min return to 0.033 Hz (upper right panel). Roman numerals refer to time points in 9A (lower panel). Lower panel shows time course of intracellular caffeine effect on Purkinje neuron EPSC_1_ amplitude. Dashed line prior to t = 0 indicates period immediately following breakthrough to whole-cell configuration, but prior to establishing stimulation conditions. Data are mean ± s.e.m. from 4 cells (1 animal). B) Representative recordings of Purkinje neuron EPSC with 30 mM BAPTA in the internal solution buffer under control conditions (upper left panel), following the addition of 50 mM caffeine (upper centre panel), and following 5 min of 1 Hz stimulation (upper right panel). Roman numerals refer to time points in 9B (lower panel). Lower panel shows time course of caffeine effect with intracellular BAPTA present on Purkinje neuron EPSC_1_ amplitude. Data are mean ± s.e.m. from 4 cells (2 animals).

With 15 mM caffeine in the internal solution, no change in EPSC_1_ amplitude was observed over 10 min of recording (Fig 9A). Furthermore, increasing stimulation frequency to 1 Hz led to a small increase in mean EPSC_1_ amplitude, which persisted after returning to 0.033 Hz (Fig 9A). These results are consistent with control slices untreated with caffeine (Fig 3A).

Caffeine can rapidly equilibrate across the cell membrane [36]. Consequently, the concentration of caffeine reaching the fine dendrites would be uncertain (but higher internal concentrations of caffeine may result in diffusion out of the Purkinje neuron to reach appreciable levels in the presynaptic terminals). Given this uncertainty, we next buffered intracellular calcium within the Purkinje neuron by including 30 mM BAPTA in the internal solution, a concentration sufficient to block calcium signalling in Purkinje neuron spines [37]. Under these recording conditions, addition of 50 mM caffeine to the bath solution resulted in the same characteristic changes in paired pulse ratio and depression at 1 Hz as for Purkinje neurons with standard internal solution (Fig 9B), suggesting changes in intracellular calcium within the Purkinje neuron do not contribute significantly to the observed effects of caffeine.

### The caffeine effect is reversible and not due to changes in osmolarity

**Fig 10:**
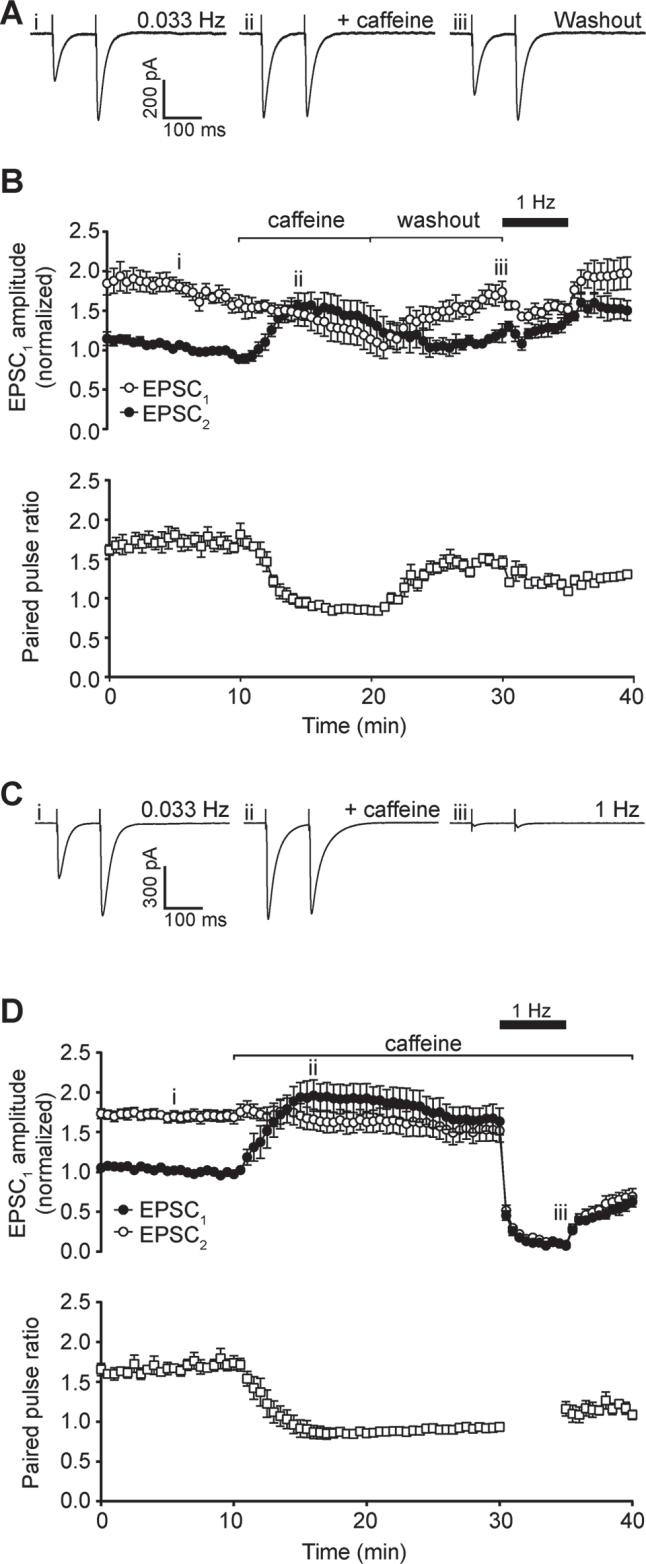
Effects of washout and osmolarity on caffeine-mediated effects. A) Representative recordings of Purkinje neuron EPSC at under control conditions (first panel), peak effect following the addition of 50 mM caffeine (second panel), and following 10 mins of wash-out (third panel). Roman numerals refer to time points in 10B. B) Time course of effect of washing out caffeine on amplitude (upper panel) of the first (filled circles, EPSC_1_) and second (open circles, EPSC_2_) pulse in each pair, and on and paired-pulse ratio (lower panel). Data are mean ± s.e.m. from 6 cells (4 animals). C) Representative recordings of Purkinje neuron EPSC at under control conditions (first panel), after addition of 50 mM caffeine in isosmotic Krebs buffer (second panel), and following 5 mins of 1 Hz stimulation (third panel). Roman numerals refer to time points in 10D. D) Time course of caffeine effect on amplitude (upper panel) of the first (filled circles, EPSC_1_) and second (open circles, EPSC_2_) pulse in each pair and on paired-pulse ratio (lower panel) after addition of isosmotic caffeine solution to bath. Data are mean ± s.e.m. from 6 cells (3 animals).

In principle, the effects of caffeine on synaptic and ectopic transmission could be a result of toxicity rather than a selective pharmacological effect, and so we next investigated the reversibility of the phenomenon. The decrease of paired-pulse ratio was shown to be reversible on wash-out, during which paired-pulse facilitation was restored to 1.46 ± 0.08 (cf. 1.65 ± 0.06 for pre-treatment; Fig 10A and 10B). Thereafter, raising stimulation frequency to 1 Hz did not cause a suppression of the evoked responses, and returning to baseline frequency elicited an overall potentiation (Fig 10B), as with untreated controls (Fig 3A).

Supplementing standard extracellular buffer with 50 mM caffeine increased bath solution osmolarity by around 10% (from 313 mOsm to 347 mOsm). As varying osmotic pressure is known to modulate release probability [38], we repeated the experiments with 50 mM caffeine prepared in a modified Krebs buffer with reduced glucose and NaCl concentrations (to 5 mM and 117 mM respectively) to match osmolarity of the standard bath solution. Reducing the osmolarity of the caffeine solution had no effect on enhancement of amplitude at 0.033 Hz or suppression at 1 Hz (Fig 10C and 10D cf. Fig 1B).

### Disrupting vesicle trafficking does not reproduce caffeine effects

The preceding results suggest that caffeine does not modulate parallel fibre transmission via any of its well-known targets. We therefore investigated other components of the vesicle recovery apparatus, speculating that the loss of inward current observed during tetanic stimulation reflects rapid depletion of the readily releasable pool (Fig 4C). In particular, previous work has shown that caffeine can disorder cytoskeletal structure in smooth muscle cells [39], which would also be predicted to disrupt vesicle recycling.

**Fig 11:**
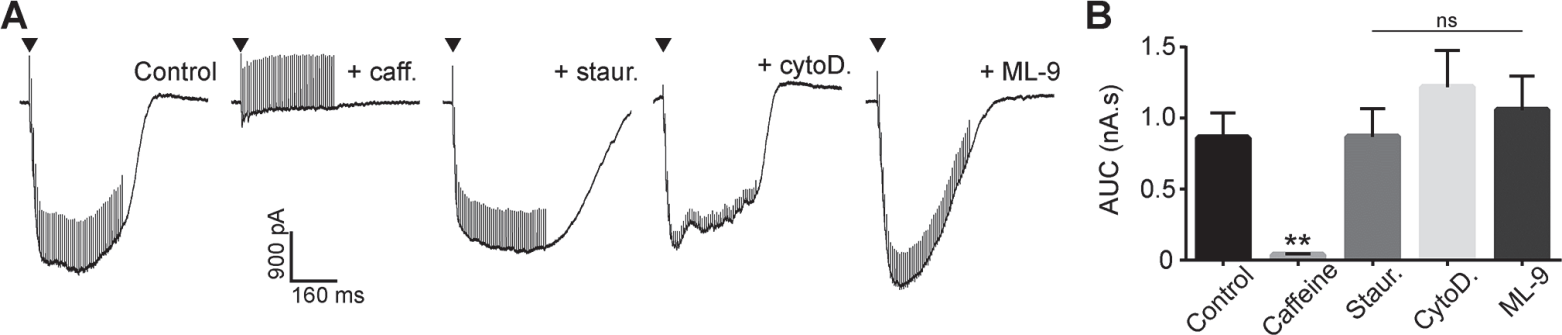
Disruption of vesicle trafficking. A) Representative whole-cell recording of neuronal EPSCs during tetanic stimulation (100 Hz, 50 pulses). Recordings are shown from the same cell before and after addition of 50 mM caffeine to bath solution. Independent recordings are next shown from cells pre-incubated with 5 μM staurosporine, 10 μM cytochalasin D, and 25 μM ML-9, respectively. Arrows indicate the initiation of tetanic stimulation. B) Area under curve (AUC) values measuring the inward current generated by tetanic stimulation. Areas were measured over a 1 s period, starting from 0.05 s prior to the onset of tetanus. Data are mean + s.e.m. from 3–8 cells, respectively, from 1–4 animals, respectively. Statistical significance was tested using one-way ANOVA with Dunnett’s Multiple Comparison test to compare each group to control. ** indicates a P value of 0.0037, ns indicates a P value of > 0.05. Slices were incubated with 2 μM DPCPX, 10 μM CGP52432, 100 μM MPPG, and 5 μM rimonabant to inhibit presynaptic inhibitory receptors.

To test this hypothesis, we pharmacologically inhibited vesicle trafficking by two mechanisms that we have previously shown to depress transmission at 1 Hz [15]: inhibition of myosin light chain kinase with ML-9 (25 μM), and inhibition of actin monofilament polymerization with cytochalasin D (10 μM). Slices were pre-incubated for 10 minutes prior to the onset of tetanic stimulation, and in both cases, bath application of the inhibitor failed to mimic the decrease in current during the tetanus observed with 50 mM caffeine (Fig 11). This suggests that despite causing similar loss of transmission at 1 Hz [15], disruption of cytoskeletal assembly and transport did not reproduce the effect of caffeine on readily releasable pool size.

We finally tested a broad-spectrum kinase inhibitor, to test whether the loss of transmission during tetanus may be due to caffeine inhibiting one of the many phosphorylation steps involved in vesicle transport, docking, priming and fusion. Staurosporine (5 μM) failed to reproduce the dramatic reduction in current observed with caffeine application (Fig 11), suggesting that if caffeine does diminish the readily releasable pool, it is not through serine/threonine phosphorylation-dependent events.

## Discussion and Conclusions

Our results show that caffeine has two effects on neurotransmitter release from parallel fibre terminals. First, paired pulse facilitation is reversed to paired pulse depression by an increase in the amplitude of the first pulse and decrease in the amplitude of the second pulse, an effect that is most dramatic at ectopic release sites. Second, caffeine led to loss of the usual potentiation of EPSC amplitude observed during 1 Hz stimulation, to instead show a progressive decrease in transmission strength—a pattern of plasticity that is closely similar to ectopic release under normal conditions.

### Effects of caffeine are not due to known pharmacological targets

Caffeine has been extensively studied as a stimulant, and is known to act at many molecular targets in the central nervous system. Perhaps the best known pharmacological effects are antagonism of adenosine receptors [40], agonism of ryanodine receptors [20], and inhibition of PDE [34]. The effects of caffeine observed in this study have a similar concentration-dependence to these known targets (and many studies employ concentrations in the 10–50 mM range used here: e.g. [29,31,41–45]) but the evidence suggests that the effects on synaptic transmission are independent of these targets.

Pharmacological manipulation of calcium release from internal stores (Figs 5 and 6) neither reproduced nor blocked the effects of caffeine. These results are consistent with previous studies, which have reported that calcium release from internal stores has no impact on EPSC amplitude or short-term plasticity at parallel fibre synapses [30,31]. Similarly, manipulation of cAMP signalling did not alter caffeine responses (Figs 7 and 8), suggesting that antagonism of tonic adenosine signalling in the molecular layer, or inhibition of PDE in the presynaptic terminals, are unable to account for the ability of caffeine to reverse paired pulse facilitation of synaptic and ectopic responses to parallel fibre stimulation, or cause failure of synaptic transmission at 1 Hz. Furthermore, previous studies have also shown that A1 adenosine receptor antagonists have no detectable effect on transmission strength or paired pulse ratio at either synaptic or ectopic release sites [46,47]. This suggests a lack of tonic adenosine signalling in the molecular layer, suggesting that antagonism of these receptors is unlikely to account for the ability of caffeine to increase the amplitude of synaptic and ectopic responses to parallel fibre stimulation.

Finally, we tested the combination of both ryanodine receptor agonism and adenylyl cyclase activation to reproduce the predicted increase in calcium and cAMP that could result from caffeine’s multiple pharmacological actions. This resulted in a similar pattern of potentiation to forskolin alone, but with somewhat reduced amplitude. This could be explained by activation of calcium-sensitive PDE as a result of calcium release from internal stores, limiting the increase in cAMP evoked by forskolin.

### Enhancement of vesicle release by caffeine

The increase in EPSC_1_ amplitude by caffeine could be explained by either an increase in the number of release competent sites, or an increase in probability of release at existing sites. An increase in the number of sites, for example through “unsilencing” of terminals [48], would not, however, be predicted to cause the decrease in paired pulse ratio observed with caffeine.

An increase in release probability could result in facilitation switching to paired pulse depression if probability was increased to the extent that the initial stimulus caused depletion of release ready vesicles (as with the climbing fibre synapse). However, the release probability at parallel fibre terminals is uncertain, with estimates being confounded by multivesicular release [22,49], rapid replenishment of vesicles [50,51], and different outcomes for stimulation of unitary and clustered inputs [52]. Furthermore, increasing release probability by either increasing extracellular calcium or increasing presynaptic cAMP does not eliminate paired pulse facilitation, adding to the evidence that these terminals retain the capacity for facilitation even if EPSC_1_ amplitude is substantially increased, mostly likely due to active replenishment of vesicles within tens of milliseconds [51].

Deducing the mechanism of enhancement of EPSC_1_ amplitude is also complicated by the second effect of caffeine: depression during 1 Hz stimulation. The mechanisms may be inter- related, on the basis that they show similar sensitivity to caffeine (Fig 3B), and so changes in paired pulse ratio may reflect both processes occurring simultaneously.

### Effects of caffeine on maintenance of readily releasable pool

Treatment with caffeine made the parallel fibre input incapable of sustaining transmission when baseline frequency was raised to 1 Hz (Figs 1, 2 and 3). Even when returning to 0.033 Hz after 5 min at 1 Hz, the recovery of EPSC amplitude was slow (Fig 1B); indeed the time course of depression and recovery came to resemble the typical response to 1 Hz stimulation observed at ectopic release sites [19].

Stimulation of parallel fibres at 100 Hz in the presence of caffeine led to a dramatic reduction in the total inward current generated during the tetanus, and a partial loss of the fast recovery of EPSC amplitude in the post-stimulus period (Fig 4C). These results are again consistent with the idea that caffeine prevents the terminals from rapidly replenishing the readily releasable pool during repetitive stimulation.

The calcium influx that triggers vesicle release in parallel fibre terminals also activates vesicle recycling and docking mechanisms [18], meaning that during a tetanus under control conditions, depletion due to release is partially counteracted by accelerated recovery of docked and primed vesicles. If caffeine inhibited this accelerated recovery, it would account for all our observations: loss of paired pulse facilitation, rapid failure of transmission during a tetanus, reduced recovery after a tetanus, and failure of transmission during sustained 1 Hz stimulation.

Accordingly, we speculate that the observed effects of caffeine on synaptic transmission can be explained by a dual mechanism: an increase in release probability coupled with an inhibition of vesicle replenishment mechanisms.

### Transmission at ectopic sites

The potentiation of release probability by caffeine at synaptic sites is also present at ectopic sites. Indeed, the extent of potentiation of the first pulse is even greater (Fig 2), most likely reflecting the lower release probability of ectopic sites. This enhancement is however accompanied by a dramatic decline in ESC amplitude even at 0.033 Hz, which is consistent with the idea that ectopic sites lack fast vesicle recovery intrinsically and so any loss of vesicles will deplete the total ectopic pool.

The EC_50_ for enhancement of ectopic transmission (>28 mM) was greater than for enhancement of synaptic transmission (6.50 mM), suggesting that whatever target caffeine is acting on to increase release probability has differing sensitivity at ectopic and synaptic sites.

### Potential targets for caffeine

Our data show that calcium release from stores and cAMP signalling are unlikely to be the mechanisms by which caffeine alters vesicle release and recovery, indicating a previously unrecognized target or targets. One noteworthy possibility comes from the recent observation that caffeine can disrupt actin polymerisation in smooth muscle cells independently of calcium or cAMP modulation [39]. There is abundant evidence for cytoskeletal rearrangements being necessary for vesicle trafficking and recycling [16], and we have previously shown that other inhibitors of actin polymerisation can render parallel fibre synapses incapable of sustaining transmission at 1 Hz [15]. However, whilst this seems to represent a plausible hypothesis for the effects of caffeine on vesicle recovery, inhibiting key components of actin cytoskeleton remodelling was not able to mimic the effect of caffeine during tetanic stimulation (Fig 11). Furthermore, broad-spectrum inhibition of kinases also failed to reproduce the loss of transmission during a tetanus, reducing the likelihood that kinase inhibition could account for the effect. Collectively, these results show that the effects of caffeine are not directly predictable from known effects on reported molecular targets; another mode of action remains to be discovered.

### Pharmacological implications of caffeine effects

The previously unrecognized ability of caffeine to alter vesicle release probability and recovery has implications for interpretation of previous studies that have used the drug to investigate cerebellar function. For example, caffeine has previously been used as a mechanism of elevating calcium in Purkinje neurons as an approach for evoking long-term depression [53]. Our results would suggest that presynaptic effects of caffeine would complicate interpretation of this experiment. Beyond the cerebellum, if caffeine acts in a similar way at other synapses, there may be a convolution of actions on release probability and recycling both through direct mechanisms and via actions on calcium release from stores, as many other synapses do show sensitivity to store-dependent calcium signalling [54]. Consequently, the use of caffeine as a pharmacological tool for investigating these phenomena should be undertaken with caution.

Another implication of our results is that it is possible to pharmacologically enhance synaptic release but with a “ceiling effect”, where overstimulation would be counteracted by the failure to recycle readily-releasable vesicles. As such, caffeine would be acting as a stimulant at excitatory terminals that fire at relatively low rates, but a depressant at terminals that fire at high frequencies. Under these conditions, the stimulant effect would be self-limiting, and indeed may even be protective under conditions of hyperexcitability such as epilepsy or migraine. Development of new compounds with this same mechanism may thus have therapeutic potential.

## References

[1] Zucker RS, Regehr WG (2002) Short-term synaptic plasticity. Annu Rev Physiol 64: 355–405. 11826273 10.1146/annurev.physiol.64.092501.114547

[2] Chen C, Regehr WG (1997) The mechanism of cAMP-mediated enhancement at a cerebellar synapse. J Neurosci 17: 8687–8694. 9348337 10.1523/JNEUROSCI.17-22-08687.1997 PMC6573078

[3] Kaneko M, Takahashi T (2004) Presynaptic mechanism underlying cAMP-dependent synaptic potentiation. J Neurosci 24: 5202–5208. 15175390 10.1523/JNEUROSCI.0999-04.2004 PMC6729197

[4] Kaeser PS, Kwon H-B, Blundell J, Chevaleyre V, Morishita W, Powell CM, et al (2008) RIM1α phosphorylation at serine-413 by protein kinase A is not required for presynaptic long-term plasticity or learning. Proc Natl Acad Sci U S A 105: 14680–14685. 10.1073/pnas.0806679105 18799741 PMC2567150

[5] Salin PA, Malenka RC, Nicoll RA (1996) Cyclic AMP mediates a presynaptic form of LTP at cerebellar parallel fiber synapses. Neuron 16: 797–803. 8607997 10.1016/s0896-6273(00)80099-9

[6] Linden DJ, Ahn S (1999) Activation of presynaptic cAMP-dependent protein kinase is required for induction of cerebellar long-term potentiation. J Neurosci 19: 10221–10227. 10575019 10.1523/JNEUROSCI.19-23-10221.1999 PMC6782423

[7] Jacoby S, Sims RE, Hartell NA (2001) Nitric oxide is required for the induction and heterosynaptic spread of long-term potentiation in rat cerebellar slices. J Physiol 535: 825–839. 11559778 10.1111/j.1469-7793.2001.t01-1-00825.x PMC2278807

[8] Matsui K, Jahr CE (2003) Ectopic release of synaptic vesicles. Neuron 40: 1173–1183. 14687551 10.1016/s0896-6273(03)00788-8

[9] Matsui K, Jahr CE, Rubio ME (2005) High-concentration rapid transients of glutamate mediate neural-glial communication via ectopic release. J Neurosci 25: 7538–7547. 16107641 10.1523/JNEUROSCI.1927-05.2005 PMC6725396

[10] Bergles DE, Dzubay JA, Jahr CE (1997) Glutamate transporter currents in Bergmann glial cells follow the time course of extrasynaptic glutamate. Proc Natl Acad Sci U S A 94: 14821–14825. 9405697 10.1073/pnas.94.26.14821 PMC25121

[11] Clark BA, Barbour B (1997) Currents evoked in Bergmann glial cells by parallel fibre stimulation in rat cerebellar slices. J Physiol 502: 335–350. 9263914 10.1111/j.1469-7793.1997.335bk.x PMC1159553

[12] Matsui K, Jahr CE (2004) Differential control of synaptic and ectopic vesicular release of glutamate. J Neurosci 24: 8932–8939. 15483112 10.1523/JNEUROSCI.2650-04.2004 PMC6730070

[13] Bellamy TC, Ogden D (2005) Short-term plasticity of Bergmann glial cell extrasynaptic currents during parallel fiber stimulation in rat cerebellum. Glia 52: 325–335. 16078233 10.1002/glia.20248

[14] Bellamy TC, Ogden D (2006) Long-term depression of neuron to glial signalling in rat cerebellar cortex. Eur J Neurosci 23: 581–586. 16420466 10.1111/j.1460-9568.2005.04588.x

[15] Balakrishnan S, Jackson C, Russell N, Bellamy TC (2011) Ectopic release sites lack fast vesicle recycling mechanisms, causing long-term depression of neuron-glial transmission in rat cerebellum. Glia 59: 82–93. 10.1002/glia.21078 20967883

[16] Rizzoli SO, Betz WJ (2005) Synaptic vesicle pools. Nat Rev Neurosci 6: 57–69. 15611727 10.1038/nrn1583

[17] Wu L-G, Ryan TA, Lagnado L (2007) Modes of vesicle retrieval at ribbon synapses, calyx-type synapses, and small central synapses. J Neurosci 27: 11793–11802. 17978015 10.1523/JNEUROSCI.3471-07.2007 PMC6673382

[18] Neher E, Sakaba T (2008) Multiple roles of calcium ions in the regulation of neurotransmitter release. Neuron 59: 861–872. 10.1016/j.neuron.2008.08.019 18817727

[19] Balakrishnan S, Bellamy TC (2009) Depression of parallel and climbing fiber transmission to Bergmann glia is input specific and correlates with increased precision of synaptic transmission. Glia 57: 393–401. 10.1002/glia.20768 18837050

[20] McPherson P, Kim Y, Valdivia H, Knudson C, Takekura H, Franzini-Armstrong C, et al (1991) The brain ryanodine receptor: a caffeine-sensitive calcium release channel. Neuron 7: 17–25. 1648939 10.1016/0896-6273(91)90070-g

[21] Konnerth A, Llano I, Armstrong CM (1990) Synaptic currents in cerebellar Purkinje cells. Proc Natl Acad Sci U S A 87: 2662–2665. 1969639 10.1073/pnas.87.7.2662 PMC53750

[22] Foster KA, Crowley JJ, Regehr WG (2005) The influence of multivesicular release and postsynaptic receptor saturation on transmission at granule cell to Purkinje cell synapses. J Neurosci 25: 11655–11665. 16354924 10.1523/JNEUROSCI.4029-05.2005 PMC6726039

[23] Batchelor AM, Garthwaite J (1992) GABA_B_ receptors in the parallel fibre pathway of rat cerebellum. Eur J Neurosci 4: 1059–1064. 12106411 10.1111/j.1460-9568.1992.tb00132.x

[24] Dittman JS, Regehr WG (1996) Contributions of calcium-dependent and calcium-independent mechanisms to presynaptic inhibition at a cerebellar synapse. J Neurosci 16: 1623–1633. 8774431 10.1523/JNEUROSCI.16-05-01623.1996 PMC6578681

[25] Dittman JS, Regehr WG (1997) Mechanism and kinetics of heterosynaptic depression at a cerebellar synapse. J Neurosci 17: 9048–9059. 9364051 10.1523/JNEUROSCI.17-23-09048.1997 PMC6573588

[26] Neale SA, Garthwaite J, Batchelor AM (2001) Metabotropic glutamate receptor subtypes modulating neurotransmission at parallel fibre–Purkinje cell synapses in rat cerebellum. Neuropharmacology 41: 42–49. 11445184 10.1016/s0028-3908(01)00046-6

[27] Brown SP, Brenowitz SD, Regehr WG (2003) Brief presynaptic bursts evoke synapse-specific retrograde inhibition mediated by endogenous cannabinoids. Nat Neurosci 6: 1048–1057. 14502290 10.1038/nn1126

[28] Bezprozvanny I, Bezprozvannaya S, Ehrlich B (1994) Caffeine-induced inhibition of inositol (1, 4, 5)-trisphosphate-gated calcium channels from cerebellum. Mol Biol Cell 5: 97–103. 8186468 10.1091/mbc.5.1.97 PMC301012

[29] Saleem H, Tovey SC, Molinski TF, Taylor CW (2014) Interactions of antagonists with subtypes of inositol 1,4,5-trisphosphate (IP3) receptor. Br J Pharmacol 171: 3298–3312. 10.1111/bph.12685 24628114 PMC4080982

[30] Sabatini B, Regehr W (1995) Detecting changes in calcium influx which contribute to synaptic modulation in mammalian brain slice. Neuropharmacology 34: 1453–1467. 8606793 10.1016/0028-3908(95)00129-t

[31] Carter AG, Vogt KE, Foster KA, Regehr WG (2002) Assessing the role of calcium-induced calcium release in short-term presynaptic plasticity at excitatory central synapses. J Neurosci 22: 21–28. 11756484 10.1523/JNEUROSCI.22-01-00021.2002 PMC6757598

[32] Simpson PB, Nahorski SR, Challiss R (1996) Agonist-evoked Ca^2+^ mobilization from stores expressing inositol 1, 4, 5-trisphosphate receptors and ryanodine receptors in cerebellar granule neurones. J Neurochem 67: 364–373. 8667014 10.1046/j.1471-4159.1996.67010364.x

[33] Peppiatt CM, Collins TJ, Mackenzie L, Conway SJ, Holmes AB, Bootman MD, et al (2003) 2-Aminoethoxydiphenyl borate (2-APB) antagonises inositol 1, 4, 5-trisphosphate-induced calcium release, inhibits calcium pumps and has a use-dependent and slowly reversible action on store-operated calcium entry channels. Cell Calcium 34: 97–108. 12767897 10.1016/s0143-4160(03)00026-5

[34] Boswell-Smith V, Spina D, Page CP (2006) Phosphodiesterase inhibitors. Br J Pharmacol 147: S252–S257. 16402111 10.1038/sj.bjp.0706495 PMC1760738

[35] Balezina O, Bukiya A (2001) Spontaneous activity of murine neuromuscular junctions in the presence of dantrolene. Neurophysiology 33: 79–85.

[36] O'Neill S, Donoso P, Eisner D (1990) The role of [Ca^2+^]_i_ and [Ca^2+^] sensitization in the caffeine contracture of rat myocytes: measurement of [Ca^2+^]_i_ and [caffeine]_i_ . J Physiol 425: 55–70. 2213589 10.1113/jphysiol.1990.sp018092 PMC1189837

[37] Coesmans M, Weber JT, De Zeeuw CI, Hansel C (2004) Bidirectional parallel fiber plasticity in the cerebellum under climbing fiber control. Neuron 44: 691–700. 15541316 10.1016/j.neuron.2004.10.031

[38] Rosenmund C, Stevens CF (1996) Definition of the readily releasable pool of vesicles at hippocampal synapses. Neuron 16: 1197–1207. 8663996 10.1016/s0896-6273(00)80146-4

[39] Tazzeo T, Bates G, Roman HN, Lauzon A-M, Khasnis MD, Eto M, et al (2012) Caffeine relaxes smooth muscle through actin depolymerization. Am J Physiol Lung Cell Mol Physiol 303: L334–L342. 10.1152/ajplung.00103.2012 22683573 PMC3774218

[40] Ribeiro JA, Sebastiao AM (2010) Caffeine and adenosine. J Alzheimers Dis 20: 3–15. 10.3233/JAD-2010-100246 20164566

[41] Alonso MT, Chamero P, Villalobos C, Garcia-Sancho J (2003) Fura-2 antagonises calcium-induced calcium release. Cell Calcium 33: 27–35. 12526885 10.1016/s0143-4160(02)00179-3

[42] Hagenston AM, Rudnick ND, Boone CE, Yeckel MF (2009) 2-Aminoethoxydiphenyl-borate (2-APB) increases excitability in pyramidal neurons. Cell Calcium 45: 310–317. 10.1016/j.ceca.2008.11.003 19100621 PMC2869079

[43] Hegg CC, Irwin M, Lucero MT (2009) Calcium store-mediated signaling in sustentacular cells of the mouse olfactory epithelium. Glia 57: 634–644. 10.1002/glia.20792 18942758 PMC2657191

[44] Montero M, Alonso MT, Carnicero E, Cuchillo-Ibanez I, Albillos A, Garcia AG, et al (2000) Chromaffin-cell stimulation triggers fast millimolar mitochondrial Ca^2+^ transients that modulate secretion. Nat Cell Biol 2: 57–61. 10655583 10.1038/35000001

[45] Morton-Jones R, Cannell M, Housley G (2008) Ca^2+^ entry via AMPA-type glutamate receptors triggers Ca^2+^-induced Ca^2+^ release from ryanodine receptors in rat spiral ganglion neurons. Cell Calcium 43: 356–366. 17719086 10.1016/j.ceca.2007.07.003

[46] Takahashi M, Kovalchuk Y, Attwell D (1995) Pre-and postsynaptic determinants of EPSC waveform at cerebellar climbing fiber and parallel fiber to Purkinje cell synapses. The Journal of Neuroscience 15: 5693–5702. 7643211 10.1523/JNEUROSCI.15-08-05693.1995 PMC6577621

[47] Bellamy TC (2007) Presynaptic modulation of parallel fibre signalling to Bergmann glia. Neuropharmacology 52: 368–375. 17011600 10.1016/j.neuropharm.2006.08.009

[48] Cousin MA, Evans GJ (2011) Activation of silent and weak synapses by cAMP-dependent protein kinase in cultured cerebellar granule neurons. J Physiol 589: 1943–1955. 10.1113/jphysiol.2010.200477 21486806 PMC3090596

[49] Bender VA, Pugh JR, Jahr CE (2009) Presynaptically expressed long-term potentiation increases multivesicular release at parallel fiber synapses. J Neurosci 29: 10974–10978. 10.1523/JNEUROSCI.2123-09.2009 19726655 PMC2775459

[50] Crowley JJ, Carter AG, Regehr WG (2007) Fast vesicle replenishment and rapid recovery from desensitization at a single synaptic release site. J Neurosci 27: 5448–5460. 17507567 10.1523/JNEUROSCI.1186-07.2007 PMC6672343

[51] Valera AM, Doussau F, Poulain B, Barbour B, Isope P (2012) Adaptation of granule cell to Purkinje cell synapses to high-frequency transmission. J Neurosci 32: 3267–3280. 10.1523/JNEUROSCI.3175-11.2012 22378898 PMC6622027

[52] Isope P, Barbour B (2002) Properties of unitary granule cell→ Purkinje cell synapses in adult rat cerebellar slices. J Neurosci 22: 9668–9678. 12427822 10.1523/JNEUROSCI.22-22-09668.2002 PMC6757845

[53] Kimura T, Sugimori M, Llinás RR (2005) Purkinje cell long-term depression is prevented by T-588, a neuroprotective compound that reduces cytosolic calcium release from intracellular stores. Proc Natl Acad Sci U S A 102: 17160–17165. 16278299 10.1073/pnas.0508190102 PMC1287999

[54] Bouchard R, Pattarini R, Geiger JD (2003) Presence and functional significance of presynaptic ryanodine receptors. Prog Neurobiol 69: 391–418. 12880633 10.1016/s0301-0082(03)00053-4

